# Effect of bundling auricular acupressure and fluid restriction program on salivary flow rate and fluid control adherence among children undergoing hemodialysis: a randomized control trial

**DOI:** 10.1186/s12912-025-03433-x

**Published:** 2025-07-01

**Authors:** Zohour Ibrahim Rashwan, Marwa Mohamed Mahmoud Abdellah, Ferial Moursi, Esraa Mohammed Abd El-Samie Ismail

**Affiliations:** 1 Pediatric Nursing, Faculty of Nursing, Alexandria University, Alexandria, Egypt; 2 Nursing Education, Faculty of Nursing, Alexandria University, Alexandria, Egypt; 3 Pediatric Nephrology Unit Alexandria, Faculty of Medicine, Alexandria University, Alexandria, Egypt

**Keywords:** Acupressure, Auricular, Fluid control, Hemodialysis, Salivary excretion, Therapeutic adherence and compliance

## Abstract

**Background:**

The adverse effect of chronic kidney disease extends far beyond the primary impact on kidney function. Children undergoing Haemodialysis (HD) develop several oral complications such as decreased salivary flow rate and xerostomia. This creates a vicious cycle where the compulsive urge to drink further contributes to fluid overload. Providing a standalone program may not be sufficient to ensure full adherence to fluid restrictions. Auricular Acupressure (AA) induces parasympathetic stimulation which increases salivary flow rate and alleviate persistent feeling of thirst.

**Aim:**

to assess the effect of bundling AA and fluid restriction program on salivary flow rate and fluid control adherence among children undergoing HD.

**Methods:**

A single-blinded randomized control trial was conducted at Alexandria University Children’s Hospital in Smouha, Egypt. A sample of 60 children receiving HD was randomly assigned to two parallel groups. The control group received the fluid restriction program through general small-group discussion sessions, individualized consultations, and follow-up. The study group also received the intervention bundle: the fluid restriction program and twelve sessions of AA on four acupoints: Shenmen, Kidney Concha, Point Zero, and Upper Tragus.

**Results:**

Subjective assessment revealed significant differences in all the parameters of salivary flow rate, such as difficulty in swallowing (*p* = 0.005), saliva rate in mouth (*p* = 0.016), dryness in mouth (0.009), dryness in lips (*p* = 0.002). The objective assessment of the salivary flow rate unveiled that applying the intervention bundle resulted in a notable increase in salivation level (from 33.3 to 86.7%) with *p*-value > 0.001 compared to a slight increase in the control group (23.3–36.7%). There was a significant improvement in the overall fluid control adherence in both groups with favor to the study group who received the intervention bundle as *p*-values were < 0.001and 0.047 respectively.

**Conclusion:**

Bundling AA and fluid restriction programs effectively improved salivary flow rate and fluid control adherence among children undergoing HD attending the HD unit in Alexandria University Children’s Hospital at Smouha, Egypt. Therefore, nurses working in HD units may adopt this intervention bundle as a cost-effective, safe, and complementary tool to promote sustainable patient adherence to fluid-restriction regimen.

**Trial registration number:**

[NCT06562959], ClinicalTrails.gov, Retrospectively registered (April 8th, 2024), URL of trial registry record: https://clinicaltrials.gov/study/NCT06562959.

## Introduction

Chronic Kidney Disease (CKD) is a devastating illness with many long-term consequences. The worldwide prevalence of CKD among children from 5 to 12 years was roughly estimated as 15-74.7 cases per million children [1, 2]. The deterioration of kidney function in CKD can eventually lead to end-stage kidney disease (ESKD) [1, 2]. The ESKD is an irreversible condition where the kidneys have been permanently and severely damaged with a glomerular filtration rate of less than 15 mL/min/1.73 m², indicating complete or near-complete loss of kidney function [1, 3]. For managing ESKD, children may require kidney transplantation or renal replacement therapy including Hemodialysis (HD), to artificially filter the blood, remove waste products, and maintain fluid and electrolyte balance. The HD sessions are typically required 3–4 times per week, for several hours each [4].

The adverse effect of ESKD extends far beyond the primary impact on kidney function. Children with ESKD undergoing HD develop several oral complications predominantly hyposalivation or decreased salivary flow [5]. Alarmingly, the high levels of creatinine and blood urea nitrogen in children with ESKD can also wreak havoc on the delicate salivary glands, leading to debilitating consequences [6]. As these waste products accumulate, they can induce atrophy and irreversible fibrosis within the salivary gland tissues, causing a reduction in saliva flow that leads to the characteristic sensation of dry mouth (xerostomia). This salivary gland dysfunction has a profoundly negative impact on children’s daily lives, severely hindering their ability to eat, speak, and swallow [7]. Consequently, they experience a persistent feeling of thirst and a compulsive urge to drink fluids. This creates a vicious cycle where the excess fluid intake further contributes to fluid overload [8].

Children with ESKD on HD demonstrate dysregulation of intravascular fluid homeostasis, leading to chronic fluid volume excess. Besides, the uncontrolled fluid intake can then exacerbate an increased interdialytic weight gain (IDWG) and strain the already compromised kidneys [9]. Excessive IDWG is a major risk factor for increased blood pressure and other potentially life-threatening complications. For these reasons, the National Kidney Foundation (2020) recommends a combined use of fluid control methods for (a) tracking fluid intake, (b) limiting sodium consumption, (c) alleviating xerostomia, (d) providing personalized fluid goals and (e) facilitating adherence to fluid-restriction [10].

Improving adherence to fluid restriction remains a cornerstone of ESKD management protocols among children. A primary factor that contributes to non-adherence with prescribed diet and fluid restriction is inadequate knowledge, negative attitude, and uncontrolled behaviour [11]. So, the pediatric nurses play a critical role in providing essential and current information regarding the specified treatment plan for children HD.

Fluid restriction poses a significant challenge and dilemma for children undergoing HD. This challenge revolves around finding ways to quench thirst while adhering to the prescribed fluid restriction regimen [12]. In order to help these children, overcome such challenges, nurses may adopt various non-pharmacological interventions. These interventions include sugar-free chewing gum or lozenges, sucking ice, saliva substitutes, mouthwash, transcutaneous electrical nerve stimulation, and Auricular Acupressure (AA) [13, 14].

Auricular acupressure is a non-invasive therapeutic technique that involves the application of pressure or stimulation to specific points on the outer ear (auricle). The AA is a form of ear acupuncture or auriculotherapy, which is based on the principle that the outer ear is a microsystem representing the entire body. It involves the application of gentle and firm pressure, usually with small beads, pellets, or magnets, over specific acupoints on the ear with a finger by licensed practitioners [15, 16] The roots of AA lie in the rich traditions of ancient healing practices, primarily within traditional Chinese medicine, which is further enriched by influences from Indian Ayurveda and ancient Egyptian medicine, which views the ear as a microsystem reflecting the entire body [15]. Bridging ancient knowledge with contemporary practice, AA has modern foundations in the work of Dr. Paul Nogier, the father of ear acupressure, who precisely mapped ear points to corresponding body organs [15]. Dr. Paul Nogier (1950) originally generated the concept of an inverted fetus map on the external ear where the head is located in the ear lobe [15, 16]. This suggests that by stimulating these specific ear points, one can potentially influence the corresponding organs and promote healing. The underlying mechanism of AA is the stimulation of the parasympathetic nervous system that innervates the salivary glands. This parasympathetic stimulation may help increase salivary flow rate and alleviate xerostomia in children on HD. Also, it increases the peripheral blood flow, which may stimulate saliva production [17].

Unlike adults, children undergoing HD face several challenges that complicate their treatment, particularly in adhering to fluid and dietary restrictions. Their developmental stage makes it difficult to fully understand and commit to the strict guidelines. This can result in serious health complications, such as fluid overload. Although there are various experimental studies and clinical trials were conducted to alleviate the symptoms of ESKD such as dry mouth and reducing thirst, most of these researches were confined to adult populations. This limits their generalizability to children and leaves a significant gap regarding the effective strategies tailored to meet the unique needs of children. Moreover, most of the existing studies tend to examine AA and fluid restriction strategies independently. As such, this study investigates the combined effect of the educational programs along with AA for improving adherence of pediatric patients with ESKD and enhances their knowledge, attitude, and behavior regarding the fluid restriction. This study also could pave the road for developing intervention bundles by adding AA into the standard treatment protocols for children undergoing HD and ultimately transforming clinical practices for these vulnerable groups to enhance their overall well-being [18].

## Study aim

This study aims to assess the effect of bundling auricular acupressure (AA) and fluid restriction program on salivary flow rate and fluid control adherence among children undergoing HD.

### Research hypothesis

Children undergoing HD who receive both AA and fluid restriction programs exhibit better salivary flow rate and fluid control adherence than those who receive fluid restriction programs only.

## Methods

### Design and settings

A single-blinded randomized control trial was conducted at the HD Unit in Alexandria University Children’s Hospital in Smouha, Egypt.

### Participants

A sample of 60 children who fulfilled the following criteria: age ranged from 6 to 18 years and receiving HD for at least one month. In contrast, the exclusion criteria included diabetes mellitus, heart diseases, and autoimmune disorders such as Sjogren’s syndrome. Moreover, children who are receiving treatment for xerostomia and mouth dryness were also excluded. The sample size was determined using G*Power 3.1.9.4 for the difference between two independent variables Power of 95%, an alpha set at 5%, an effect size of 0.9, and allocation N2/N1 = 1 [19, 20]; the sample size required for the former analysis was 56 (28 for each group). A dropout rate of 20% was anticipated; thus, we aimed to recruit an additional 12 participants.

After obtaining the ethical approval as well as the permission from the responsible administrative authorities to conduct the study, the medical records of all children on HD were accessed to get the sample. A list of the eligible children who fulfilled the inclusion criteria and agreed to participate in the study (sampling frame) was obtained. Participants were randomly allocated to one of two balanced study arms: the study group (received the intervention bundle, fluid restriction program, and AA) and the control group (received the fluid restriction program only).

**Fig. 1:**
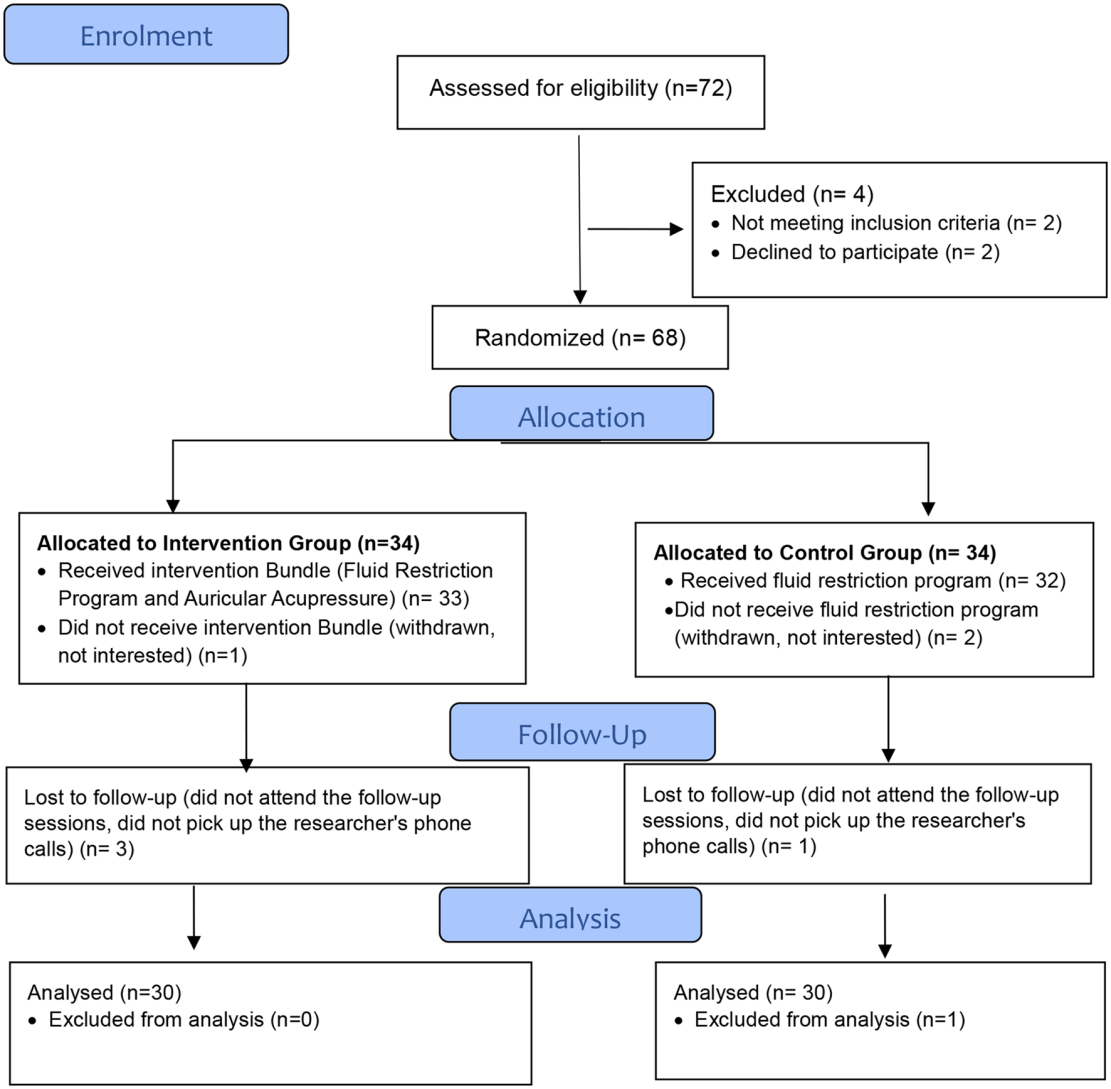
Consort Flow Chart of Participants’ Allocation

For concealment of the allocation process, the block randomization method with a block size of 4 was used where one participant was assigned to the study group, and the next one was assigned to the control group with a 1:1 ratio (Fig. 1) [21]. The randomization sequences were created by an independent party (professional statistician) who was not involved in the research processes. The randomization sequence of the participants in both groups was kept in a secure place in two separate sealed opaque envelopes. As for the allocation concealment, the envelopes were opened just before initiating the clinical trial. The researchers also kept the data assessor and statistical analysts blinded [21, 22]. The data were collected by two volunteer nurses who helped the research team distribute the questionnaire blindly without knowing the participant allocation and the study hypothesis. The biostatistician who performed the data analysis was not informed about the groups’ assignment [22]. Additionally, the research supervisors (ZIR) closely monitored the entire process of randomization and masking to ensure its proper implementation.

### Measurements tool

Initially, the researcher assessed children’s socio-demographic characteristics (e.g., age, gender, residence) and clinical data (date of the first HD session, frequency and duration of HD sessions).

### Salivary flow visual analogue scale

This tool was adopted by Pai et al. (2001) to assess the salivary flow rate. It consisted of 8 subjective items measuring two main dimensions of salivary flow [23]. The first dimension assesses impaired oral functions due to the sensation of dry mouth, as indicated by difficulty swallowing or speaking (items 1 and 2). The second dimension evaluates the dryness of oral mucosa (lips, mouth, tongue, or throat) (items 4, 5, 6, and 7). Additionally, children were asked two global questions regarding salivary quantity and thirst level (items 3 and 8). In each item, children’s responses were rated on a 100-mm horizontal numeric scale ranging from No (0) to very dry (100). The higher score indicated increasing dryness and low salivary flow rate. The scale reliability ranged from 0.27 ~ 0.83 [23]. Moreover, Pai et al. (2001) evaluated the validity of the VAS questionnaire by comparing its baseline scores with various baseline salivary measures during a second visit. They found significant correlations between five VAS items and USFR. Additionally, they analysed the correlations of VAS responses with SPFR and SSFR over a 6-hour placebo trial to assess the stability of these responses [23].

**Table:** 

Items	Scoring
1 and 2	Not difficult at all (0) to very difficult (100)
3	A lot (0) to None (100)
4,5,6,7	Not dry at all (0) to very dry (100)
8	Not thirsty at all (0) to very thirsty (100)

### Modified schirmer test

This test consisted of strips with rounded notched tape. In order to objectively assess the salivary flow rate, the strips had a clear graduation from 5 to 35 mm. Typically, the strips exhibited a color change from white to blue when they came in contact with the oral saliva. The salivary flow reading was determined by the length of the colored change on the strip into three main levels: (a) hyposalivation (reading less than 15 mm after 3 min of contact with the saliva), (b) normal salivation (reading more than 15–34 mm in 1 min) and (c) hypersalivation (reading is 35 mm in 1 min) [24].

### Fluid control in HD patients scale (FCHPS)

This scale was developed by Cosar and Pakyuz (2016) to evaluate the knowledge, behavior, and attitude toward fluid restriction among patients with HD [25]. The FCHPS includes items measuring patients’ compliance with fluid control. It consisted of 24 items under three main subscales, namely, Knowledge (items 1–7), Behavior (items 8–18) and Attitude (items 19–24). Each item is rated on a three-point Likert ranging from “3 = agree”, “2 = indecisive “, and “1 = don’t agree”. The scale included positive and negative items, whereas the negative items (6, 7, and 18–24) were reverse-scored. The total score ranges from 24 to 72. The higher the score, the higher the patients’ adherence to fluid control. Çoşar and Pakyuz. (2016) assessed both the validity and reliability of the FCHPS scale. The content validity was conducted by the panel of expert who reviewed and approved the scale items. In addition, the explanatory factor analysis of Çoşar and Pakyuz (2016) revealed three sub-dimensions including knowledge, behavior and attitude with a total of 24 items, explaining a total variance of 51.15%. According to Çoşar and Pakyuz. (2016) the scale was reliable where the Cronbach’s alpha reliability coefficient was 0.88 for the overall questionnaire and 0.92, 0.80, and 0.67 for the subdimensions respectively. The test and retest correlation revealed 0.94 (*p* < 0.001), indicating strong reliability [25].

### Data collection procedure

#### The data was collected through four phases as follows

##### Assessment phase

Initially, the searchers approached the participants one day before the first session and asked them to refrain eating, drinking, or brushing their teeth for 2 h before the attending the sessions. The data collections were done by two trained volunteer nurses who were blinded about the study hypothesis and concealed the participants’ group allocation. As part of the baseline data, children in both groups were assessed for socio-demographic characteristics, clinical data, salivary flow rate, and adherence to fluid control, which included their knowledge, attitudes, and behaviors regarding the fluid restriction regimen. Additionally, baseline unstimulated salivary flow rate was measured before the first session at 9 am. Participants were instructed to sit in an upright position, swallow all saliva beforehand, and not to swallow again while the strips were applied. The strip was placed on the floor of the mouth, near the submandibular gland duct (adjacent to the lingual frenum), and results were recorded based on the length of wetting after 3 min.

##### Preparation and planning phase

In the preparation phase, the researchers developed the fluid restriction program’s educational objectives and content. The program is designed to achieve four main objectives: (a) Explain a fluid-restricted regimen, (b) Describe measures for alleviating dryness of mouth and thirst sensation, (c) Illustrate techniques for fluid intake self-monitoring, and (d) Empower fluid-restricted regimen adherence.

In order to design the fluid restriction program content (booklet), the researcher thoroughly reviewed related literature and the current evidence, and a focus group with five expert nurses in the HD unit was conducted. The booklet included five main elements as follows;

- ***Fluid distribution guidelines***: Children are advised to spread out fluid intake evenly throughout the day rather than consuming large volumes at once. The booklet also provided practical advice on measuring and tracking their fluid intake using a measuring cup, calculating the consumed fluid (e.g., 1 ounce (oz) = 30 ml = 2 tablespoons; 1 cup juicy fruit/veg = ½ cup fluid; 1 cup ice cubes/chips = ½ cup fluid melted). Children are also equipped with essential tips for documenting fluid intake, including those fluids used in taking medications.
- ***Identification of fluid-rich foods***: The booklet offered a detailed list of foods that contain high amounts of water and should be counted as part of a patient’s fluid intake. This included fruits like berries, watermelon, and grapes, as well as desserts and other items like popsicles, soups, sauces, gravies, puddings, yogurt, and salad dressings. It also covers foods that melt into liquid, such as gelatin, ice cream, and milkshakes.
- ***Sodium-restricted diet***: The program emphasized the importance of a sodium-restricted diet for children on HD (i.e., less than 2 g (or 2000 mg) sodium daily). It advised patients to avoid canned, smoked, and heavily processed foods, which tend to be high in sodium. Patients were instructed to check nutrition labels and choose low-sodium options. The program also recommended using herbs instead of salt.
- ***Thirst management techniques***: The booklet provided various strategies to help quench thirst and alleviate dry mouth. Suggestions included keeping hard candies, mints, and sugar-free gum on hand, as well as techniques like brushing teeth, using chilled mouthwash, and sucking on measured amounts of ice cubes, lemon slices, or frozen fruit. Adding lemon or cucumber to water was also recommended to make water more palatable and thirst-quenching. Children were also advised to breathe through their noses rather than their mouths to reduce insensible water loss.
- ***Weight monitoring***: The program emphasized the importance of daily tracking of the body weight using the same scale. Patients were instructed to report any abnormal weight gain, defined as more than 3 pounds, to their healthcare provider to address fluid overload issues.

To check the content validity of the final version of the fluid restriction program, a panel of experts (*n* = 6), including two nephrologists, one pediatrician, and three specialized nephrology nurses, was invited to review it and examine its scientific content and clarity. Based on the panel recommendations and feedback, many amendments, additions, and deletions were made. The overall agreement was 0.86 for relevancy and 0.91 for clarity [21].

Teaching sessions for groups were planned according to participants’ pre-scheduled sessions, and the researchers arranged with the HD supervisors to utilize the meeting room for delivering the sessions. The intervention started in June 2023 and ended in February 2024.

##### Implementation phase

Children of both groups received the fluid restriction program at week zero while children in the study group received additional 12 sessions of AA over four weeks. The first session of the AA was started in the following week of the fluid restriction program to ensure that the cumulative effect of the AA sessions enhances the body’s response to fluid restriction and promotes salivary gland activity.

**Fluid Restriction Program** included (a) General small-group discussion sessions, (b) individualized consultations, and (c) follow-up sessions.

I. ***Group Discussion Sessions***

In the group discussion sessions, children in both the study and control groups were divided into subgroups (6 participants per each). Initially, the researchers introduced themselves, established rapport with children and their caregivers, and distributed the educational booklet. Then, the sessions were delivered by one of the researcher, who holds a Ph.D. in Pediatric nursing and has extensive experience in children with ESKD. The sessions were conducted in the HD unit’s meeting room, lasting 45 min each. Eventually, children were instructed to follow the fluid-restricted regimen guided by the booklet.

II. ***Individualized Consultations***

After the general group discussion session, the researchers conducted a comprehensive assessment to understand the child’s current fluid intake, dietary habits, and any factors that may impact their adherence. They then provided tailored, age-appropriate education to the children and their caregivers on the importance of fluid management and strategies for implementation. Collaboratively, the researchers worked with children and caregivers to develop a realistic fluid management plan, setting individualized realistic goals and identifying tools to help in tracking their fluid intake.

III. ***Follow-up***

The researchers also arranged for regular follow-up for one month i.e., during the subsequent HD sessions and by conducting daily communication through social networking applications and telephone calls to ensure their commitment to the program, monitoring the children’s progress, discussing any evolving issues and providing the needed support.

### Auricular acupressure

**Fig. 2:**
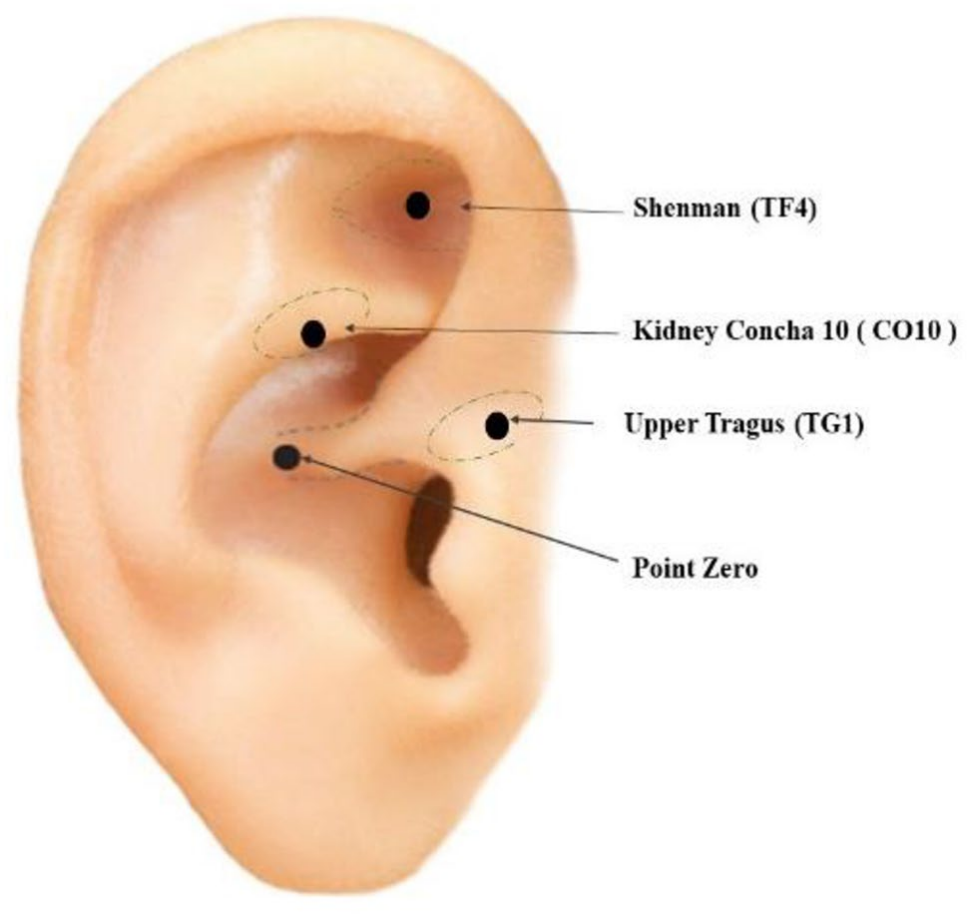
Auricular Acupoints

**The study group** received the fluid restriction program along with AA as follows;

The researcher determined the following four Acupoints (Fig. 2). These Acupoints were selected based on previous studied [26, 27].

- Shenmen Point (Triangular Fossa, TF4): located at the bifurcation of the crura of antihelix in the ear in the upper part of the posterior 1/3 of the triangular fossa (i.e., the triangular fossa four region, TF4).
- Kidney Concha 10 (CO10): located on the back of the bottom ear helix.
- Upper Tragus (TG1) Acupoint: located on the upper 1/2 of the external tragus.
- Point Zero: located in the center of the ear. The During the HD session, the researchers performed acupressure at all the previously mentioned acupoints [26]. The researcher’s finger gently pressed each determined acupoint in a circular movement clockwise for three minutes. The intervention was discontinued immediately in the presence of any signs of distress, such as eye squeezing, brow contraction, grimacing, or mouth opening. The acupressure was applied three times a week for four weeks, for a total of twelve sessions [26].

### Evaluation

Similar to the baseline data, the unstimulated salivary flow rate was measured at 9 am during the last session, the salivary flow rate as well as fluid control adherence was re-assessed by the same nurses.

### Ethical consideration

Ethical approval was obtained from the Ethical Research Committee at the Faculty of Nursing, Alexandria University, to conduct the study (AU-20-4-226-IRB00013620), and the study was retrospectively registered on clinicalTrails.gov (NCT06562959). Additional permission was obtained from the responsible authorities for the study setting after explaining the study’s aim. The study was conducted in accordance with the principles of the Declaration of Helsinki, 7th revision [28]. Before applying the interventions, the eligible children and their caregivers received a detailed explanation of the aim, benefits, and any possible risks of the study. The researchers emphasized voluntary participation and the right to withdraw from the study at any time. Participants’ privacy and anonymity, as well as the confidentiality of the obtained information, were ascertained. Accordingly, written informed consent /assent were obtained and signed by a legally authorized representative (children’s guardians).

### Statistical analysis

The Statistical Package for Social Sciences (SPSS) version 23 was used to analyze the data. Descriptive statistics, including numbers, percentages, means, and standard deviations, were calculated to summarize the demographic characteristics, salivary flow rate, and fluid control adherence. The Kolmogorov-Smirnov test was used to assess the normality of the study variables, which revealed that the data was not normally distributed. Fisher’s exact test was employed to examine the significance of differences in socio-demographic and clinical data between the study and control groups. For between-group comparisons of salivary flow rate and fluid control adherence, the Mann–Whitney U test was utilized. To compare the scores within the groups before and after the intervention, the Wilcoxon Signed Ranks test (Z) was used. All the statistical analyses were considered significant at a 2- sided *p*-value *≤* 0.05 [21].

## Results

**Fig. 3:**
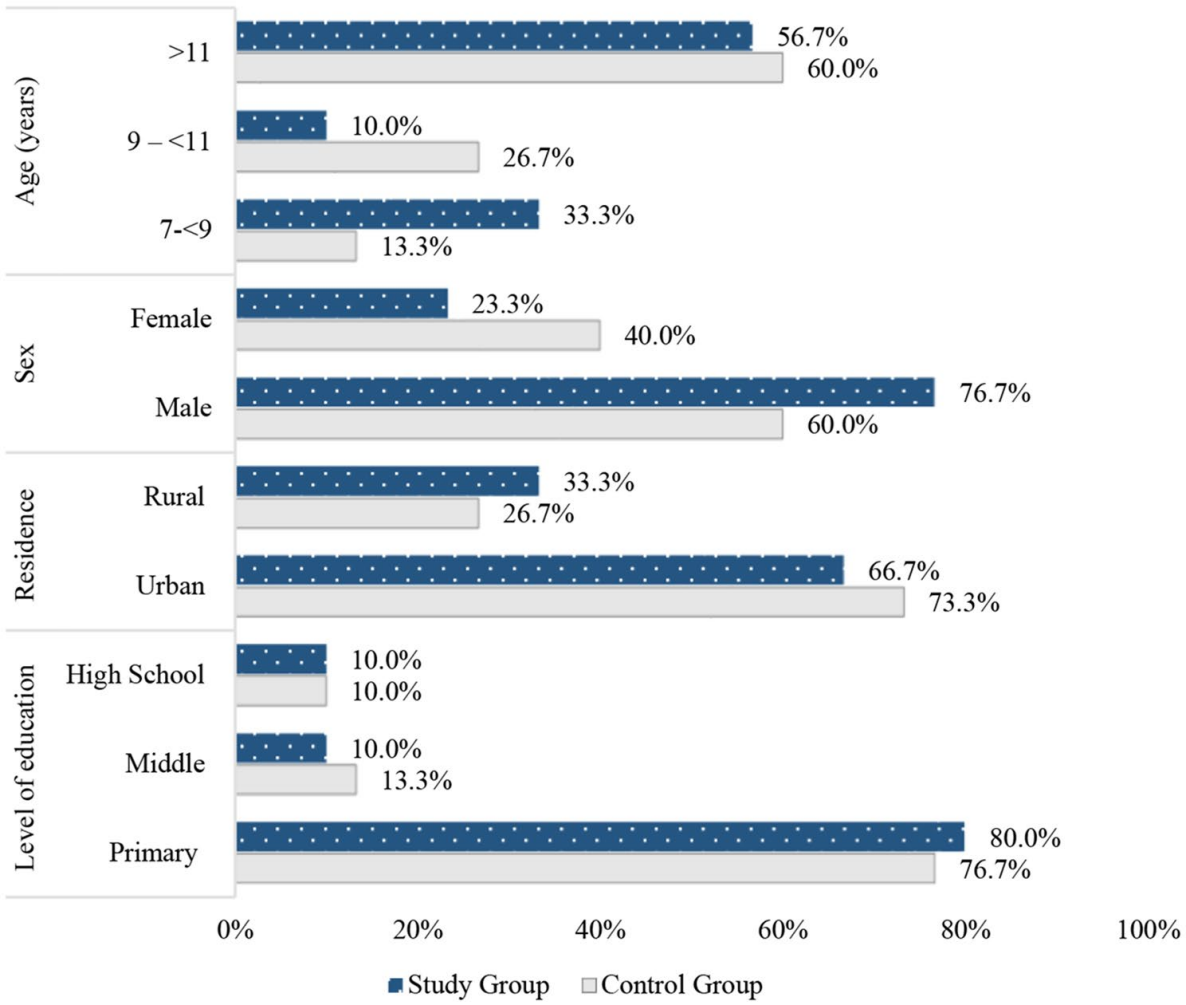
Percentage distribution of socio-demographic characteristics of children Undergoing HD

Figure 3 shows that 56.7% of children in the study group, compared to 60% of children in the control group, were at the age of 11–18 years old. Male children represented 76.7% and 60% of the study and control group respectively. Children in both groups were homogeneous as no significant statistical differences were found.

**Fig. 4:**
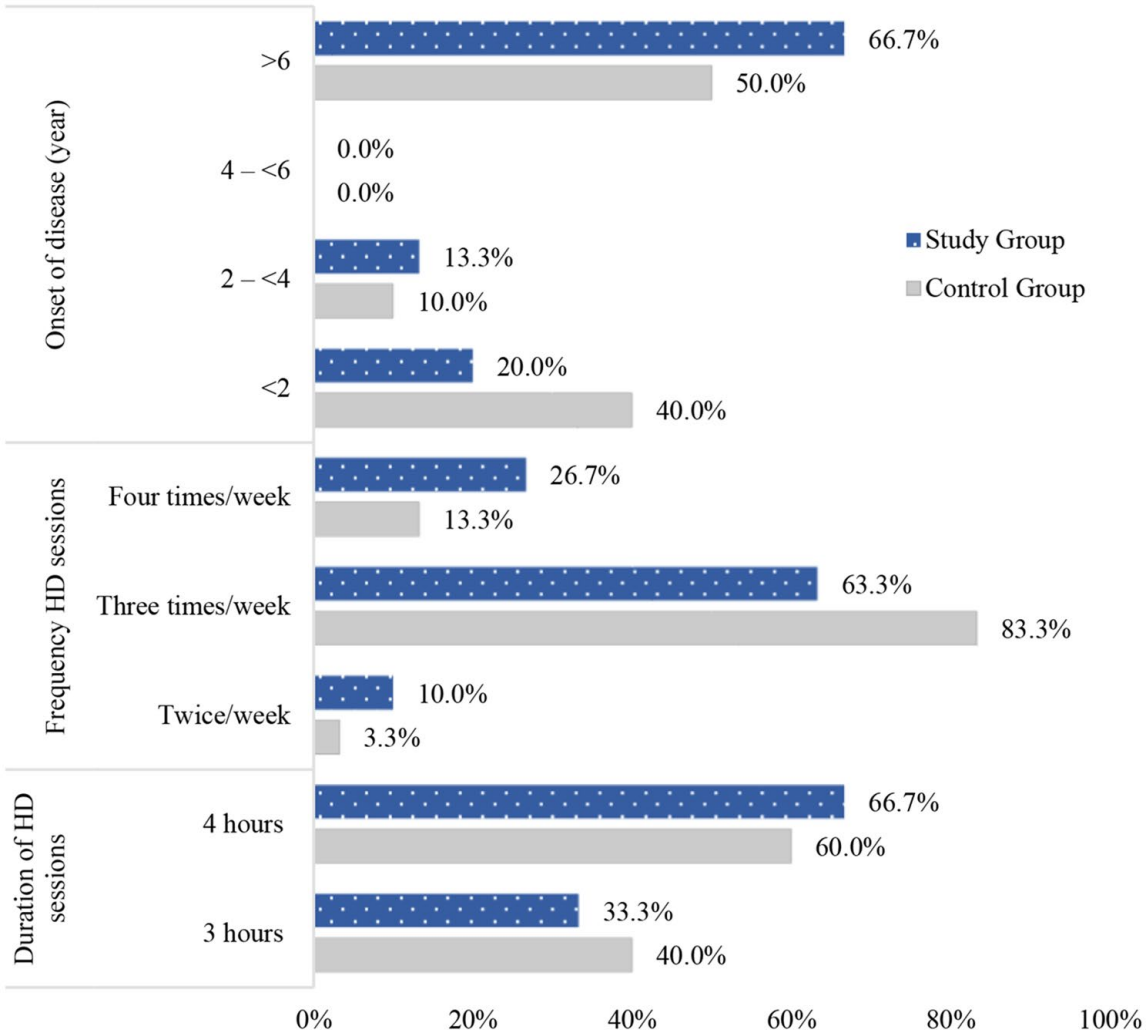
Percentage distribution of medical data of children undergoing HD

Figure 4 reveals that 66.7% of children in the study group and 50% of children in the control group were diagnosed with CKD more than six years ago. Regarding the frequency of HD sessions, it is clarified that 63.3% and 83.3% of children in the study and control groups, respectively, received HD sessions three times per week that lasted four hours (66.7% and 60% of the two groups). There is no significant statistical difference between the two groups with regards to the clinical data.

Table 1 displays a Comparison between Mean Scores of Subjective Salivary Flow Rate Assessment among children undergoing HD. There was a statistically significant difference in all the salivary flow rate parameters, including difficulty in speaking (*p* = 0.002), difficulty in swallowing (*p* = 0.005), saliva rate in mouth (*p* = 0.016), dryness in mouth (0.009), dryness in lips (*p* = 0.002) compared to only significant differences in the dryness in lips and thirst level as *p* = 0.017 and *p* = 0.001 respectively. Likewise, there was a significant improvement in overall scores on the salivary flow visual analogue scale in the study group (*p* < 0.001) compared to the control group (*p* = 0.403). In contrast, there was no statistically significant difference in the control group before and after applying the fluid restriction program.

**Table 1:** Comparison between mean scores of subjective salivary flow rate assessment among children undergoing HD

Scales	Control Group (*n* = 30)	Study Group (*n* = 30)	*p*-Value
	Mean ± SD	Mean ± SD	
**1- Difficulty in speaking**			
Before	74.0 ± 16.3	77.0 ± 13.4	**0.440**
After	69.7 ± 11.3	60.3 ± 18.1	**0.020** ^***a***^
Significance	**0.244**	**0.001** ^***b***^	
***2-Difficulty in swallowing***			
Before	66.3 ± 13.5	68.0 ± 23.0	**0.382**
After	62.7 ± 23.9	53.3 ± 19.7	**0.004** ^***b***^
Significance	**0.456**	**0.015** ^***a***^	
***3-Saliva Rate in mouth***			
Before	69.0 ± 18.3	70.0 ± 22.7	**0.474**
After	65.3 ± 27.3	52.7 ± 21.3	**0.002** ^***b***^
Significance	**0.554**	**0.016** ^***a***^	
***4- Dryness in mouth***			
Before	76.0 ± 15.2	81.3 ± 16.3	**0.129**
After	70.3 ± 14.7	66.7 ± 18.4	**0.399**
Significance	**0.040** ^*a*^	**0.011** ^*a*^	
***5-Dryness in throat***			
Before	73.7 ± 20.3	80.0 ± 17.6	**0.201**
After	67.0 ± 15.8	64.0 ± 23.9	**0.568**
Significance	**0.136**	**0.005** ^***b***^	
***6-Dryness in lips***			
Before	78.0 ± 13.5	82.7 ± 16.4	**0.223**
After	68.1 ± 18.0	55.3 ± 18.5	**0.010** ^***a***^
Significance	**0.024** ^***a***^	**0.001** ^***c***^	
***7-Dryness in tongue*** Before			
Before	73.3 ± 14.5	57.0 ± 28.8	**0.117**
After	64.0 ± 10.5	52.7 ± 18.0	**0.006** ^***b***^
Significance	**0.035** ^***a***^	**0.421**	
***8-Thirst level***			
Before	82.0 ± 14.5	75.7 ± 24.2	**0.224**
After	68.7 ± 18.1	57.3 ± 15.7	**0.012** ^***b***^
Significance	**0.002** ^***b***^	**0.003** ^***b***^	
***Total score***			
Before	72.3 ± 10.3	74.0 ± 10.4	**0.526**
After	59.3 ± 9.5	57.8 ± 8.9	**< 0.001** ^***c***^
Significance	**0.034** ^***a***^	**< 0.001** ^**c**^	

*U*: Mann-Whitney U-test *Z* = Wilcoxon Signed Rank ^a^*p*<0.05, ^*b*^*p*<0.01, ^*c*^*p*<0.001

Table 2 illustrates significant improvement among children in a study group in their knowledge, behavior, and attitude after the intervention bundle as *p*-values were 0.003, 0.001, and < 0.001, respectively. However, there were improvements in children’s knowledge and attitude toward fluid adherence in the control group after the implementation of the fluid restriction program as *p* = 0.045 and 0 0.014. There is a significant increment in the overall FCHPS in both groups in favor of the study group.

**Table 2:** Comparison between control and study groups according to knowledge, behavior, and attitude about fluid control in children undergoing HD

Fluid control	Control Group (*n* = 30)	Study Group (*n* = 30)	Significance
	Mean ± SD	Mean ± SD	
**1-Knowledge**			
*Before* *After*	9.33 ± 1.58 10.57 ± 1.22	9.20 ± 1.52 11.83 ± 4.20	**0.740** **0.008** ^***b***^
***Significance***	**0. 045** ^**a**^	**0.003** ^***b***^	
***2-Behavior***			
*Before*	15.07 ± 2.95	15.67 ± 2.51	**0.399**
*After*	16.43 ± 4.35	19.0 ± 5.09	**0.040** ^**a**^
***Significance***	**0.111**	**0.001** ^***b***^	
***3-Attitude***			
*Before*	7.53 ± 1.36	7.80 ± 1.21	**0.426**
*After*	8.70 ± 1.88	10.57 ± 3.65	**0.017** ^**a**^
***Significance***	**0.014** ^**a**^	**< 0.001** ^***c***^	
***Overall FCHPS***	31.93 ± 3.69	32.6 ± 3.36	**0.424**
	35.70 ± 5.50	41.40 ± 7.65	**0.001** ^***b***^
***Significance***	**0.047** ^**a**^	**< 0.001** ^***c***^	

*U*: Mann-Whitney U-test *Z* = Wilcoxon Signed Rank ^a^*p*<0.05, ^*b*^*p*<0.01, ^*c*^*p*<0.001

**Fig. 5:**
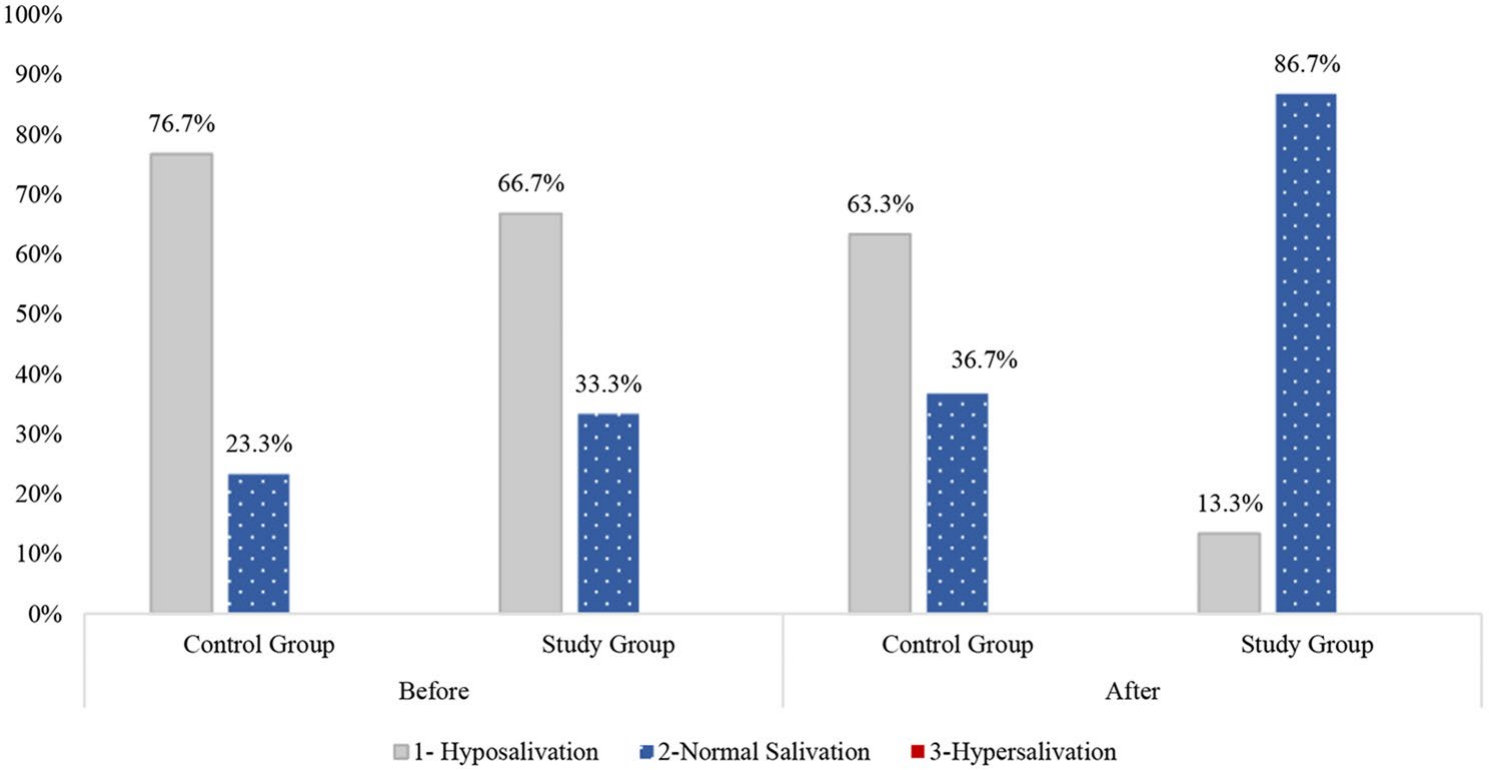
Comparison between mean scores of objective salivary flow rate assessment among children undergoing HD

The objective assessment of salivary flow rate using the Modified Schirmer Test revealed that applying the intervention bundle (AA and fluid restriction program) resulted in a notable increase in salivary flow rate (from 33.3 to 86.7%) with a *p*-value < 0.001 compared to a slight increment among children in the control group (from 23.3 to 36.7%), and the *p*-value was 0.125, as illustrated in Fig. 5.

Table 3 shows that children in both groups had hyposalivation rate before the intervention where the mean score of salivary flow among children in the control group was 12.23 ± 1.43 compared 13.27 ± 1.48 (t = 0.850, *p* = 0.399) in the study group. Despite both groups showed improvement of the salivary flow rate toward normal, the study group had higher mean score (23.88 ± 3.35) compared to slight increase in the control group (16.36 ± 2.49) with significant statistical differences between the two groups (t = 4.566, *p* < 0.001). The table also demonstrates a large effect size of all the subscales of salivary flow rate among children at the study and control groups at post-test where the Cohen’s d values was 1.726 reflecting a practical significance of the intervention bundle.

**Table 3:** Comparison between control and study groups according to salivary flow rate using modified schirmer test in children undergoing HD

Modified Schirmer Test	Pre-test	Post-test	Significance
	Mean ± SD	Mean ± SD	
Control Group (*n* = 30)	12.23 ± 1.43	16.36 ± 2.49	Z = 0.368, *p* = 0.502
Study Group (*n* = 30)	13.27 ± 1.48	23.88 ± 3.35	Z = 2.234, *p* < 0.001*
*Significance*	**U = 0.850**, ***p*** **= 0.399**	**U = 4.566**, ***p*** **< 0.001**^**c**^	
Effect Size -Cohen’s d		**1.726**	

*U*: Mann-Whitney U-test *Z* = Wilcoxon Signed Rank ^*c*^*p*<0.001. Cohen’s d: Little or no effect (0–0.2), Small effect size S (0.2–0.5), Medium effect size M (0.5–0.8), Large effect size L (0.8 or more) (Cohen, 1988)

## Discussion

Oral pathological conditions are widely observed in children undergoing HD. Dryness in oral mucosa is a frustrating symptom and manifests a frequent compelling urge to consume water or other fluids [27]. These contribute to inadequate adherence to the fluid restriction regimen and thereby increase fluid overload that potentially may result in severe cardiovascular complications. This is evident in the results of the current study, where most of the children in both the study and control groups reported low levels of compliance with fluid control. Similarly, Baryakova et al., (2023) confirmed that lack of understanding about dietary restrictions, forgetfulness, and the perception that the restrictions are onerous and identified as significant barriers to maintaining adherence to the prescribed treatment regimens [29]. Asumta et al. (2024) identified several factors that collectively contribute to non-adherence to fluid restriction observed in patients with CKD undergoing HD. These factors include socio-demographic characteristics, the perceived level of control over the disease and susceptibility as well as their knowledge about their condition and treatment [30].

Educating patients with chronic diseases, particularly ESKD, about their condition and treatment not only enhances their knowledge but also promotes their active participation in their own care [31]. This, in turn, led to better adherence to treatment recommendations and improved overall health outcomes. To better navigate the complexities of maintaining optimal adherence throughout the course of treatment, vigilant nurses should put extra effort into enhancing children’s compliance with the given instructions. In this regard, Chang et al. (2021) suggested employing empowerment strategies, such as (a) enhancing patients’ self-control through fluid intake tracking, (b) providing patient-centered education to improve disease-related knowledge, (c) offering skill-building through support groups, and (d) facilitating social support, can effectively change fluid intake behaviors among patients with CKD [26]. This ultimately leads to reduced IDWG as a key indicator of adherence to fluid restrictions in this population. Besides, Ellano et al. (2024) affirmed that the fluid distribution timetable intervention had a notable impact in reducing thirst levels among the experimental group participants during weeks three and four of their trial compared to the control group who received the standard care [32]. In a similar vein, our findings reflect a significant improvement in children’s knowledge about the significance of dietary and fluid management as well as their attitudes and behaviors towards fluid restrictions. Such improvement may be attributed to the detailed program about (a)fluid distribution guidelines, (b)identification of fluid-rich foods, (c)sodium-restricted diet, (d)thirst management techniques, and (e)weight monitoring [32]. This is in addition to the tailored individualized consultation sessions for managing each child’s unique needs and problems. In the current study, the researcher also kept in touch with the children to empower their adherence to the given instructions. In congruence with our findings, Beerappa et al. (2019) found that patients who received dietary counselling were more likely to comply with the dietary restrictions, underscoring the importance of patient education and support in fostering obedience to dietary advice [33]. In a multi-center parallel blinded cluster-randomized controlled trial conducted by Griva et al. (2018), 134 patients undergoing maintenance HD at 14 dialysis centers in Singapore received four sessions of self-management educational intervention (HED-SMART) using the principles of problem-solving and social learning theory. The results of this research reflected a significant improvement in the patient’s clinical outcomes, such as self-reported adherence, self-management skills, and reduced IDWG, in the first week, three months, and nine months post-intervention [34].

The findings of baseline assessment in the current study revealed that most children reported dryness in their oral mucosa in addition to high levels of thirst before intervention. Yang et al. (2017) asserted that such dryness sensation is not only solely attributed to salivary gland dysfunction among children on HD but also may be exacerbated by certain xerostomizing medications such as antihypertensive drugs that may lead to hyposalivation. Given the multifaceted nature of this issue, several studies recommended the adoption of nursing interventions to manage these common problems [27].

Since providing a standalone program may not be sufficient to ensure full adherence to the challenging restrictions required by these vulnerable children, this study investigates the potential of a complementary non-pharmacological nursing intervention to alleviate the debilitating oral symptoms among children on HD [26]. The AA is a practical intervention for relieving thirst and dryness in the mouth, which is considered the most prevalent oral pathological condition among patients with ESKD undergoing HD. The findings of the current study revealed a significant reduction in both subjective and objective salivary flow assessment among children in the study group compared to the control one. Children who received both AA and fluid-restriction adherence programs reported less difficulty in swallowing and speaking as well as lower sensation of dryness in their tongue, mouth, lips, and throat. Objectively, the Modified Schirmer Test revealed a significant reduction in hyposalivation (less than 15 mm after 3 min of contact with the saliva). These positive results may be attributed to the potential effects of bundling AA and fluid restriction programs that enhanced children’s adherence to fluid restrictions. In our study, the health educational program was coupled with one of the enabling interventions that increased the salivary flow rate and decreased the dryness sensation, which, in turn, empowered children to comply with the instructions. Undoubtedly, AA alleviates pathway blockages, rebalances energy flow, and restores normal bodily functions [35]. Moreover, AA has the capability to activate the autonomic nervous system, thereby triggering the release of various neuropeptides such as vasoactive intestinal polypeptide, neuropeptide Y, substance P, calcitonin gene-related peptide, and neurokinin A, leading to increased saliva production to alleviate thirst and dryness in mouth [36]. Another viewpoint proposed by Jung and Chang (2024) indicates that applying pressure on specific acupoints on the ear and neck over a four-week period can modulate the vagus nerve and increase peripheral blood flow, resulting in a significant rise in salivary flow rate among patients on HD [37]. This assertion finds support in the research conducted by Yıldırım Keskin & Tasic (2021), who investigated the impact of acupressure on thirst severity and quality of life in patients with HD. Their study revealed that acupressure was associated with enhanced saliva production, reduced thirst severity, and a positive influence on the patient’s overall quality of life [38].

A similar study conducted by Chang et al. (2021) revealed that the combined use of AA and a fluid-restriction adherence program had beneficial effects on improving salivary flow rate, fluid control, interdialytic weight gain (IDWG), and dialysis-related quality of life (DQOL) in patients on HD [26]. The significant improvements were maintained for 22 weeks after the intervention, indicating that adding AA to a fluid-adherence program had a stronger and longer-lasting impact on improving fluid control and quality of life compared to only providing a fluid-restriction program alone. The authors suggest that healthcare professionals working in HD units could consider adopting this combined AA and fluid-adherence program approach as a cost-effective, safe, and complementary strategy to help HD patients achieve more sustainable adherence to fluid restriction recommendations [26]. Besides, Jung and Chang (2024) cited that AA has both biological and clinical plausibility with regard to the treatment of dry mouth. In agreement with the result of the present study, this research showed that acupressure significantly reduced xerostomia, alleviated the negative symptoms of xerostomia for four weeks, and improved the quality of life in patients with HD [37].

On the other hand, the study of Yang et al. (2017) was incongruent with the current study, as they reported that acupressure had no effect on IDWG in chronic HD patients [27]. Moreover, Yang et al. (2010) mentioned that the salivary flow rate did not increase progressively during the first, second, and third weeks after applying the acupressure period; the salivary flow rate only increased after acupressure treatment completion. These results also suggest that acupressure may require at least twelve sessions (three sessions per week for four weeks) to reach the ‘threshold’ where the effect is present [39].

### Strengths and limitation of study

Even though the current study showed a favourable impact of the AA and fluid restriction program, the small size of the sample used in the research may affect the generalizability. However, children in both groups were homogenous after applying the inclusion and exclusion criteria. Despite the limited number of children attending the HD unit, the researchers overcome this limitation by randomly assigning cases to the study and control groups. The duration of the HD sessions offered an excellent opportunity for the researchers to spend more time providing individualized consultation and detailed explanations of the treatment plan. The researchers also benefit from the mandatory IDWG assessment prior to the HD sessions to identify children’s progress. Another strength of this study is allowing caregivers to accompany their children while attending the educational sessions, which may contribute to better adherence to the fluid restriction regimen. Nonetheless, the short follow-up period of just four weeks might hinder researcher’s ability to evaluate the long-term benefits of the interventions, suggesting that extended follow-up could provide a clearer insight upon the sustainable impact of the interventions. Furthermore, the use of single blind approach and cultural factors may also affect the generalizability of the results. Addressing these limitations in future research would strengthen the findings and provide a more confirmation of the interventions’ effectiveness.

#### Conclusion

Bundling AA and fluid restriction programs had a valuable effect on improving salivary flow rate and fluid control adherence among children undergoing HD attending the HD unit in Alexandria University Children’s Hospital at Smouha, Egypt. Therefore, pediatric nurses working in HD units may adopt this program as a cost-effective, safe, and complementary tool to promote sustainable patient adherence to fluid-restriction regimen.

### Practice implications

The pediatric nurses play a pivotal role in improving the quality of life of children undergoing hemodialysis, alleviate their suffering and manage symptoms. Improving adherence to fluid restriction regimen is fundamental action for these children [9]. So, nurses must guarantee the provision of comprehensive knowledge, a constructive attitude, and regulated behaviors concerning the fluid restriction protocols [26]. In this context, nurses should develop comprehensive education programs that encompass both the fluid restriction protocol and the benefits of AA. These programs should be designed to engage both patients and their families. Hence, enhancing understanding of the rationale behind fluid restrictions and the role of AA, thereby improving their adherence rates. Additionally, educational materials should be culturally sensitive and tailored to the diverse patients [31, 32].

Individualized consultation session in the current study enabled nurses to develop tailored care plan based on every child’s unique medical history, preferences, and circumstances. The current study highlighted that providing educational session is not enough where the regular follow-ups and assessments allowed nurses to adjust these plans based on real-time feedback and ensured children compliance with the given instructions and foster their self-management behaviors [33]. So, it is essential for nurses to keep in touch with their patient especially those with chronic illness and need dietary restrictions [33].

The current study recommends integrating Auricular Acupressure (AA) into nursing practice for those children as a cost-effectiveness non-pharmacological intervention due to its ability to stimulate the parasympathetic nervous system and thereby, increases salivary flow and alleviates sensations of thirst [27]. This is particularly beneficial for pediatric patients who often experience dry mouth and related complications due to decreased salivary output. Incorporating AA enables nurses not only improve patient comfort but also directly address the challenges and fostering better adherence to fluid restrictions regimen [29, 30].

Training nursing staff on the application of AA techniques is vital for ensuring consistent and effective implementation. Workshops and hands-on training sessions can empower nurses with the necessary skills and confidence to perform AA safely and effectively. Additionally, creating a standard procedure for integrating AA into routine care can help standardize practices across the unit [26]. Collaboration among a multidisciplinary team, including dietitians, nephrologists, and nursing staff, is essential for addressing the multifaceted challenges faced by pediatric HD patients and enhances the overall quality of care provided.

## Data Availability

The datasets used and analyzed during the current study are available from the corresponding author upon reasonable request.
